# A Comparative Study on the Addition Methods of TiO_2_ Sintering Aid to the Properties of Porous Alumina Membrane Support

**DOI:** 10.3390/membranes8030049

**Published:** 2018-07-25

**Authors:** Yulong Yang, Qibing Chang, Zhiwen Hu, Xiaozhen Zhang

**Affiliations:** Department of Materials Science and Engineering, Jingdezhen Ceramic Institute, Jingdezhen 333403, China; yangyulong@jci.edu.cn (Y.Y.); huzhiwen@jci.edu.cn (Z.H.); zhangxiaozhen@jci.edu.cn (X.Z.)

**Keywords:** membrane fabrication, ceramic membrane, support, Al_2_O_3_, TiO_2_, sintering aid

## Abstract

TiO_2_ is usually used as a sintering aid to lower the sintering temperature of porous alumina membrane support. Two ways of the addition of TiO_2_ are chosen: in-situ precipitation and in-situ hydrolysis. The results show that the distribution status of TiO_2_ has an important effect on the property of porous alumina membrane support. In in-situ hydrolysis method, the nano-meter scale TiO_2_ distributes evenly on the alumina particles’ surface. The bending strength of the support increases sharply and the pore size distribution changes more sharply along with the content of TiO_2_ which slightly increases from 0.3 wt.% to 0.4 wt.%. The distribution of the nano-meter scale TiO_2_ is not so even added by in-situ precipitation method. Neither the bending strength nor the pore size distribution of the support is worse than that of the support added by in-situ hydrolysis even if the content of TiO_2_ is high to 2 wt.%. The permeating flux has a similar tendency. Consequently, the porous alumina membrane support has the porosity of 30.01% and the bending strength of 77.33 MPa after sintering at 1650 °C for 2 h with the optimized TiO_2_ content of 0.4 wt.% added by the in-situ hydrolysis method.

## 1. Introduction

Porous alumina membranes have been widely used in the fields of gas and liquid filtration, purification, separation, thermal insulation and other sides with the advantages of their high porosity, high temperature resistance, good corrosion resistance and high chemical stability [1,2,3,4]. However, porous alumina requires the sintering temperature of above 1700 °C due to its melting point is 2050 °C. Under this condition, the abnormal grain growth of alumina grains results easily in the decrease of the bending strength and the widening of the pore size distribution [5,6,7]. Generally, a suitable porosity (3540%) is preferred to the porous alumina membrane support to balance the high filtration flux and the high bending strength in practical applications. Currently, the mechanical properties of the alumina supports are improved by increasing the sintering temperature, prolonging sintering time, or using sintering aids and so on [8,9]. Under the view of engineering, it has been proved that adding a sintering aid is a simple and feasible way [10,11] which could balance the bending strength and the porosity. TiO_2_ is chosen because TiO_2_ has similar lattice parameters to Al_2_O_3_. The solid solution can be easily formed during the sintering process. At the same time, the lattice defects are generated in the solid solution due to the valence difference, which promotes the mass transport and reduces the sintering process. As a result, the required sintering temperature can be reduced if the mechanical strength can be maintained [12,13]. 

The mixing of Al_2_O_3_ and a small amount of TiO_2_ (<2 wt.%) is not uniform by ball milling, which results in a bad bending strength, porosity and wide pore size distribution [14]. In the present work, the adding of TiO_2_ by the in-situ precipitation and the in-situ hydrolysis method were chosen to make the mixing of small amount of TiO_2_ (<2 wt.%) with coarse alumina grains (ca. 29 μm). The comparative study was carried out to understand the effect of the TiO_2_ distribution on the sintering behavior and the mechanical strength of the porous alumina membrane supports.

## 2. Materials and Methods 

### 2.1. Samples Preparation

**Figure 1:**
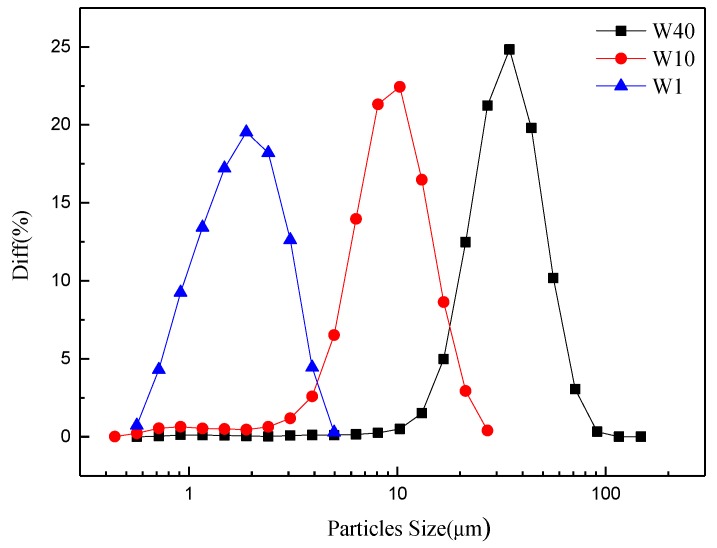
Particle size distribution of W40, W10 and W1 powders.

Alumina (α-Al_2_O_3_) powders (Luoyang, China, Purify ≥99%) were used without further treatment. The median sizes (d_50_) of the three alumina powders (coarse, medium and fine particles) are 29 μm, 8.2 μm and 1.6 μm, which are denoted as W40, W10 and W1, respectively. The particle size distributions of the three alumina powders are shown in Figure 1.

#### 2.1.1. In-Situ Precipitation Method

The addition process of TiO_2_ by in-situ precipitation method was as follows: the three kinds of particle sizes of alumina powders were mixed with a ball mill at 150 rpm for 2 h with weight ratio of 7:2.4:0.6. In the ball milling, the mass ratio of powder/alumina ball/alcohol is 1:1:1.8. The obtained suspension was dried directly in an oven at 70 °C for overnight. Urea was dissolved into water and then mixed and stirring with in Ti(SO_4_)_2_ solution (0.25 mol/L) at ice-water bathing. The mole ratio of urea/Ti(SO_4_)_2_ is 2.2:1. The urea-Ti(SO_4_)_2_ solution mixed and covered just the alumina mixture in the beaker. The beaker was moved directly into oven at 85 °C. Nano-TiO_2_ was generated based on the reaction: CO(NH_2_)_2_ + 3H_2_O → 2NH_3_·H_2_O + CO_2_↑, NH_3_·H_2_O → NH_4_^+^ + OH^−^, Ti^4+^ + 4OH^−^ → Ti(OH)_4_↓, Ti(OH)_4_ → TiO_2_ + H_2_O. After dried directly, the alumina-Ti(OH)_4_ precipitation was shaped into the rectangular bars of 30 mm × 9 mm × 5 mm (L × h × w) by dry pressing(12 MPa). Ti(OH)_4_ changed into nano TiO_2_ during the sintering without mass losses. The bars were finally sintered at 1650 °C for 2 h to form the porous alumina membrane supports. The added Ti(SO_4_)_2_ is calculated based on the nano TiO_2_ in the membrane support whose content is in the range of 0.6–2 wt.%. 

#### 2.1.2. In-Situ Hydrolysis Method

The preparation processes were similar with those in the in-situ precipitation method. The difference was that the rectangular alumina bars were firstly shaped and pre-sintered at 1350 °C. The obtained porous bars were fully immersed in the solution of tetrabutyl titanate in alcohol-water (0.7 mol/L) following the drying at 80 °C. The bars were finally re-sintered at 1650 °C for 2 h to form the porous alumina membrane supports. The nano TiO_2_ was generated based the reaction that Ti(OC_4_H_9_)_4_ + 4H_2_O → Ti(OH)_4_ + 4C_4_H_9_OH, After calcination, Ti(OH)_4_ changed into nano TiO_2_. The added Ti(OC_4_H_9_)_4_ is calculated based on the nano TiO_2_ in the membrane support whose content is in the range of 0.3–0.7 wt.%. 

### 2.2. Characterization

The particles coated by nano TiO_2_ were observed using a Transmission Electronic Microscope (TEM, JEOL-2010, Japan Electronics Co., Ltd., Tokyo, Japan). The sample was prepared by mashing the alumina grains coated by nano TiO_2_ in the mortar until the grains were suitable for TEM observation.

The pore size distribution and the porosity of the sintered compacts were measured by Mercury Intrusion Porosimetry (Autopore IV9500, Micromeritics Instrument Corporation, Norcross, GA, USA). 

The bending strength was measured by the three-point bending method at room temperature using a universal material testing machine (WDW-30, Xi’an Letry Machine Testing Co. Ltd., Xi’an, China), with a span length of 20 mm and loading speed of 0.2 mm/min. Five samples were measured and the data were averaged as the bending strength. The fracture surfaces of the sintered ceramic supports were observed by means of Field Emitting Scanning Electron Microscope (FE-SEM, JSM-6700F, JEOL, Japan Electronics Co., Ltd., Tokyo, Japan).

## 3. Results and Discussion

### 3.1. Effect of the Adding Method of TiO_2_ on the Bending Strength of Membrane Supports 

**Figure 2:**
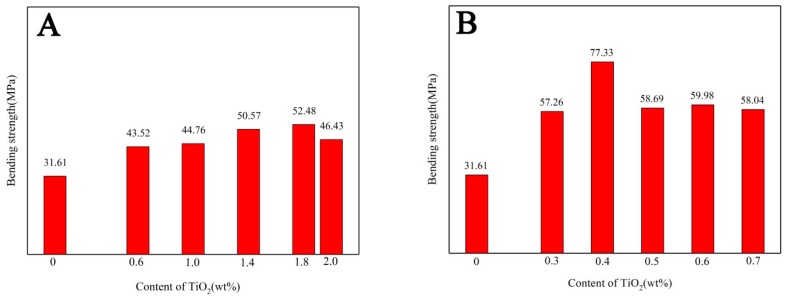
Bending strength of ceramic membrane supports with different contents of TiO_2_ by (**A**) in-situ precipitation and (**B**) in-situ hydrolysis.

**Figure 3:**
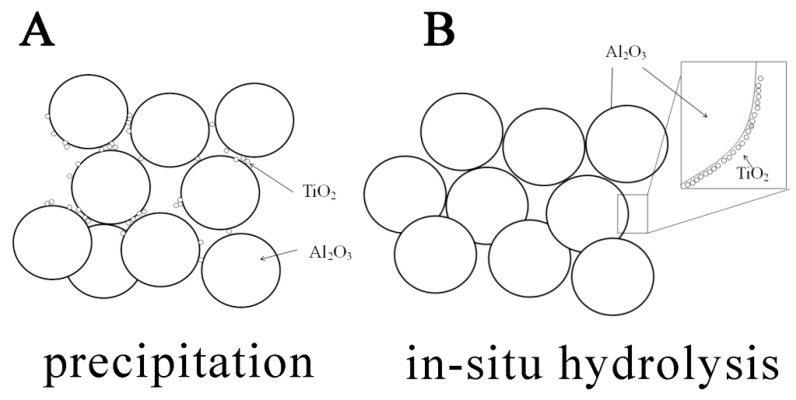
The proposed distribution of nano TiO_2_ in porous alumina supports prepared by (**A**) precipitation and (**B**) in-situ hydrolysis.

Figure 2 shows the bending strength of the porous alumina membrane supports with different contents of TiO_2_ added by in-situ precipitation and in-situ hydrolysis method. As it can be seen, the bending strength increases firstly and then decreases with the increasing of TiO_2_ content. It is verified that the addition of TiO_2_ contributes to improve the bending strength of the membrane support no matter the addition method, which is agree with our previous work [14]^.^ It can be explained that TiO_2_ reacts with alumina and forms the solid solution [15]. The fine TiO_2_ particles migrate to the alumina neck and the defect in the solid solution promotes the mass transfer to the neck area. The enlarged neck area increases the bending strength of the membrane supports without decreasing the porosity [16,17,18]. However, the support with TiO_2_ added by in-situ hydrolysis has a higher bending strength than that obtained by in-situ precipitation despite less TiO_2_ content. The difference is explained by the proposal that the uneven distribution of nano TiO_2_ in porous alumina support, as shown in Figure 3.

In precipitation process, the reaction 2CO(NH_2_)_2_ + 6H_2_O + Ti^4+^ → Ti(OH)_4_ + 4NH_4_^+^ + 2CO_2_↑ is carried quickly out in the solution and the obtained Ti(OH)_4_ is in flocculation status. In fact, alumina particles is mixed with the Ti(OH)_4_ precipitation. There is no strong interaction between alumina particles and Ti(OH)_4_. After calcination, nano TiO_2_ is also in less flocculation status and distributes randomly among alumina particles. However, in hydrolysis process, the reaction Ti(OC_4_H_9_)_4_ + 4H_2_O → Ti(OH)_4_ + 4C_4_H_9_OH is carried out step by step. The obtained Ti(OH)_4_ (sol) is free and tends to be adsorbed on the alumina particles surface. A coating maybe formed.

**Figure 4:**
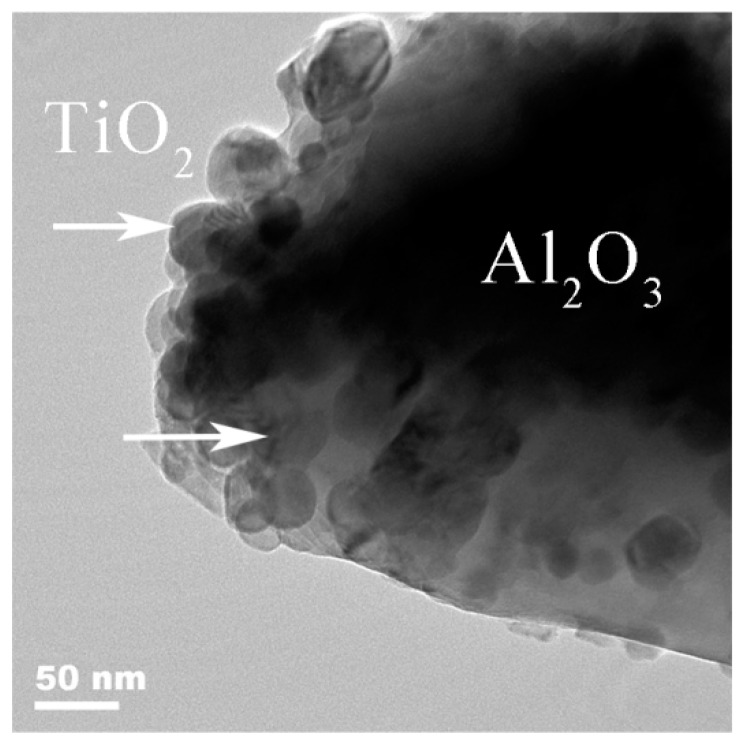
TEM image of alumina particles surface with 0.4 wt.% TiO_2_ added.

Figure 4 shows the TEM image of alumina particles surface with 0.4 wt.% TiO_2_ added. There is a nano-TiO_2_ thin layer with thickness of 50–150 nm covering the alumina particle surface, which is verified by the proposal shown in Figure 3 and is reasonable. Obviously, the hydrolysis process makes nano-TiO_2_ distributes more even. It also means that more nano-TiO_2_ act as the sintering aid. 

**Figure 5:**
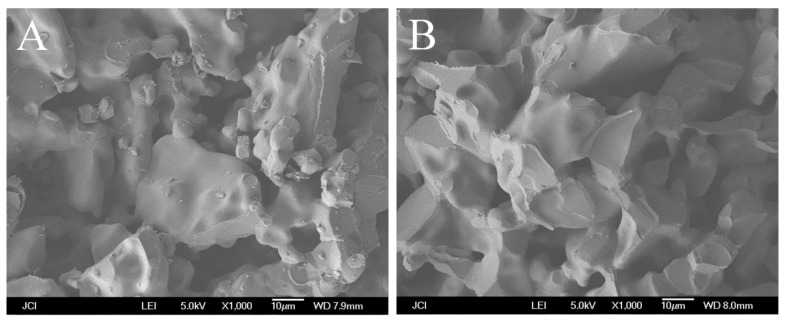
SEM images of the cross-section of supports with 0.6 wt.% of TiO_2_ added by (**A**) in-situ precipitation method and (**B**) in-situ hydrolysis method.

The bending strength is higher for the alumina support with nano-TiO_2_ added by the hydrolysis process if the TiO_2_ added is the same. Figure 5 shows the SEM images of the support with 0.6 wt.% of TiO_2_ added. As shown in Figure 5A, there are still a lot of fine TiO_2_ particles remaining over the supports with TiO_2_ added by in-situ precipitation method. However, this is not shown in Figure 5B. More importantly, many volcanic ring-like structures can be found in the fractural section. It is the remaining of the enlarged neck area of alumina grains aggregated by TiO_2_. The annular structure reflects the neck area, which has an important effect on the bending strength of the porous alumina support. The wider the neck area is, the higher the bending strength of the support. 

**Figure 6:**
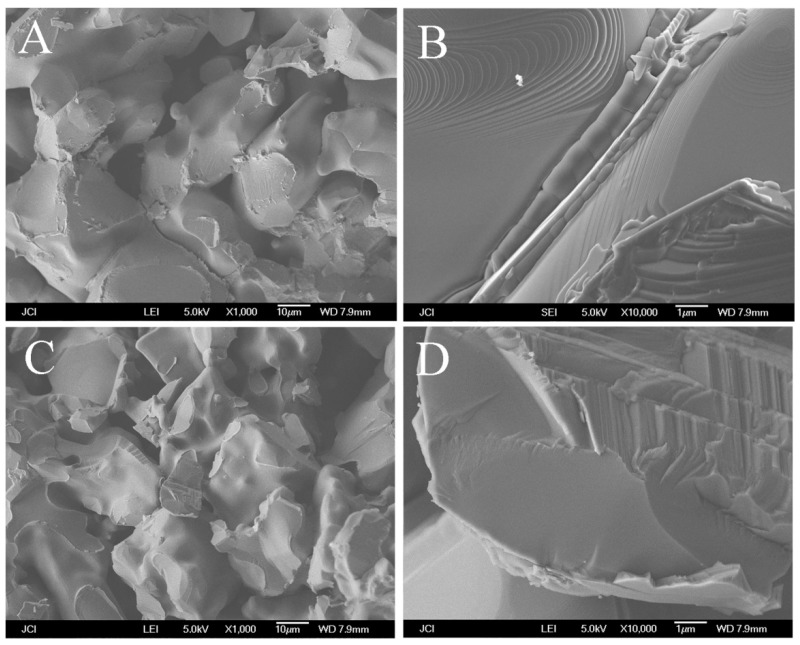
SEM images of the cross-section of the supports with (**A**,**B**) 2.0 wt.% TiO_2_ added by in-situ precipitation and (**C**,**D**) 0.4 wt.%TiO_2_ added by in-situ hydrolysis.

However, increasing the content of TiO_2_ added directly does not contribute to the bending strength of the porous alumina support. Figure 6 shows the SEM image of the supports with 2.0 wt.% TiO_2_ added by in-situ precipitation and 0.4 wt.% TiO_2_ added by in-situ hydrolysis method.

As shown in Figure 6B, some of TiO_2_ grains fill in the interspace among the porous alumina support, which decrease the porosity of the support. The TiO_2_ grains undergo the chemical reaction with Al_2_O_3_ at over 1500 °C but the decomposing again during cooling to ambient temperature. Some of TiO_2_ grains locate at the interval among the porous support and separate two alumina particles. The bending strength decreases accordingly because the theoretical strength of TiO_2_ is lower than that of Al_2_O_3_. During the cooling process, the decomposition Al_2_TiO_5_ will result in the micro-cracks among the alumina particles, which weaken the bending strength of the support. The inter-granular fracture exists in Figure 6A,B, however, the trans-granular fracture exists in Figure 6C,D with TiO_2_ added by in-situ hydrolysis, which agree with the reported by P, Monash [19]. Therefore, the distribution of TiO_2_ changes the fracture mechanism and has the effect on the bending strength of the support.

### 3.2. Effect of the Adding Methods of TiO_2_ on the Pore Size Distribution 

**Figure 7:**
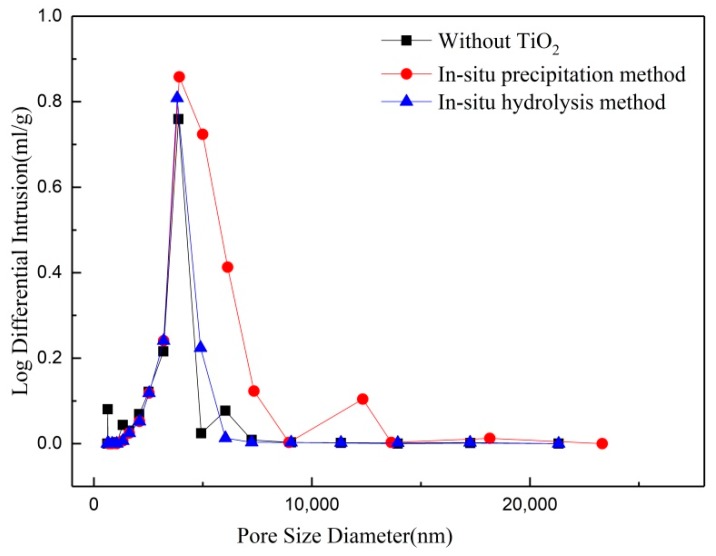
Pore size distributions of the supports with 0.6 wt.% TiO_2_ added by in-situ precipitation and in-situ hydrolysis method.

The pore size distribution of the porous membrane support depends on the particles size and the accumulation of the raw powders. For the given raw powders, the pore size distribution changes if the uniformity of the powders accumulation (pore structure for the porous ceramic) is bad. Figure 7 shows the pore size distribution of the supports with 0.6 wt.% TiO_2_ added by in-situ precipitation and in-situ hydrolysis. It can be seen that there are the similar pore size distributions no matter the adding methods, which is decided by the particle size of alumina powders. The tiny difference is that the pore size distribution of support with TiO_2_ added by in-situ precipitation is relatively wider and the curve shows a double-peak. It is reflected that the pore structure is not even. As discussed above, the uneven pore structure of the porous alumina support originates in the uneven distribution of the nano TiO_2_. 

**Figure 8:**
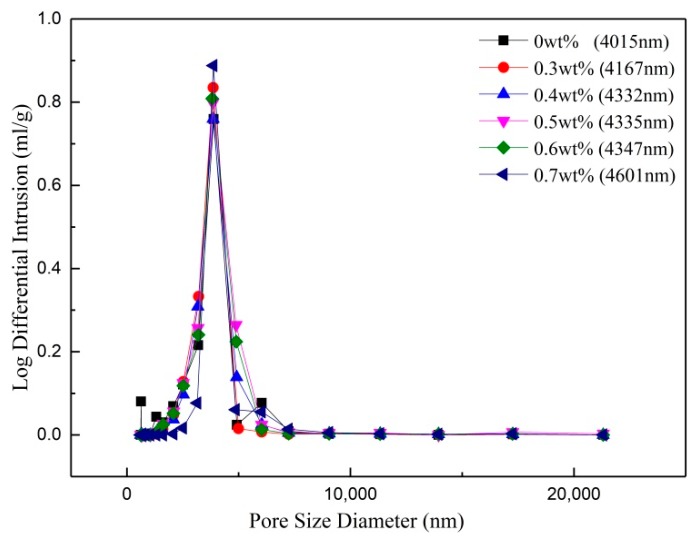
Pore size distribution of the supports with different contents of TiO_2_ added by in-situ hydrolysis method.

Figure 8 shows the pore size distribution of the supports with different contents of TiO_2_ added by in-situ hydrolysis. The pore size distributions are close to overlapping. The tiny difference is that the mean pore size increases from 4.167 μm to 4.601 μm with the increase of the TiO_2_ content. At the same time, the pore size distributions of the supports changes into narrow. It may be explained by the observation that the increased TiO_2_ enlarges the neck area and makes the pore structure sleek. The measured pore sizes change large based on mercury intrusion porosimetry because mercury can be easily intruded. 

### 3.3. Effect of the Adding Methods of TiO_2_ on the Permeating Flux 

**Figure 9:**
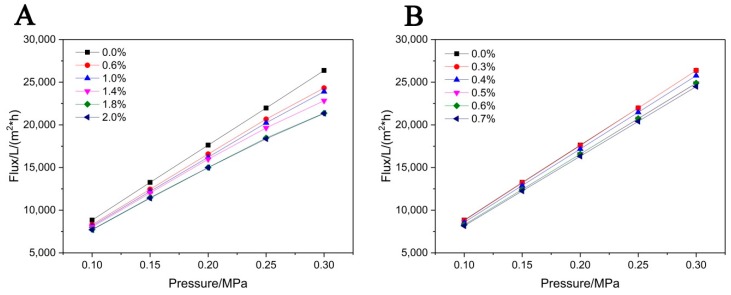
Water permeating flux of the supports with different contents of TiO_2_ added by (**A**) in-situ precipitation method and (**B**) in-situ hydrolysis method.

Figure 9 shows the water-permeating flux of the supports with different contents of TiO_2_ added by in-situ hydrolysis and in-situ precipitation method. As it can be seen, the water flux of the supports added TiO_2_ by in-situ hydrolysis has the linearly relationship with the pressure. The water permeability keeps a constant in rang of 0.10–0.3 MPa. However, the water permeability of the supports added TiO_2_ by in-situ precipitation decreases slightly with the trans-membrane pressure. It implies that the support has a high permeating resistance due to the high tortuosity [20], which is also verified by images shown in Figure 5. 

**Figure 10:**
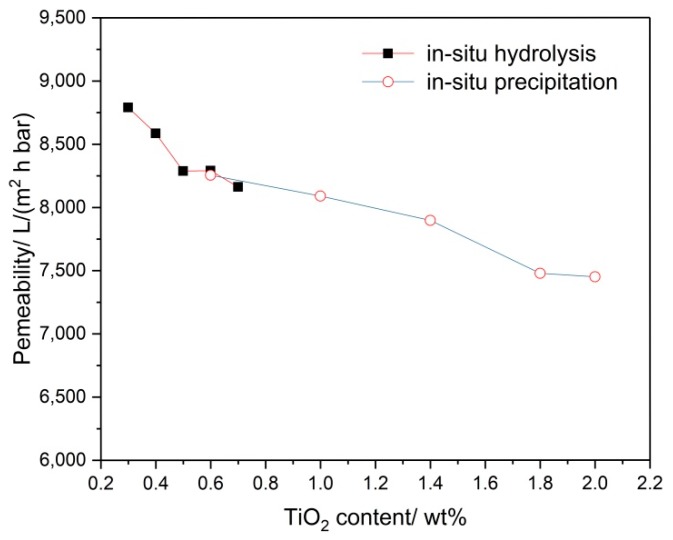
Water permeability of the supports with different contents of TiO_2_ added.

Figure 10 shows the water permeability of the supports with different contents of TiO_2_ added. Obviously, the water permeability of the supports decreases with the TiO_2_ content. The high TiO_2_ content may result in low porosity due to the action of sintering aid although it may not provide the high bending strength. The uneven distribution of the TiO_2_ particles increases the tortuosity of the porous support. Both the low porosity and the high tortuosity improve the permeate resistance according to the Hagen Poiseuille equation. Therefore, the TiO_2_ added by in-situ hydrolysis maybe the better method to prepare the membrane supports.

## 4. Conclusions

The effects of the sintering aid TiO_2_ added by in-situ precipitation and in-situ hydrolysis on the properties of the porous alumina membrane supports were comparatively investigated. The distribution status of TiO_2_ has an important effect on the property of porous alumina membrane support. The distribution of TiO_2_ added in-situ hydrolysis method is more even than that by precipitation method due to Ti(OH)_4_ (sol) adsorbed on the alumina particles’ surface. The bending strength of the support increase sharply and the pore size distribution changes more sharply along with the content of TiO_2_ slightly increasing from 0.3 wt.% to 0.4 wt.%. The porous alumina membrane support has the porosity of 30.01% and the bending strength of 77.33 MPa after sintering at 1650 °C for 2 h with the optimized TiO_2_ content of 0.4 wt.% added by the in-situ hydrolysis method. The TiO_2_ added by in-situ hydrolysis maybe a better method to prepare the membrane supports.

## References

[1] Sarkar S., Bandyopadhyay S., Larbot A., Cerneaux S. (2018). New clay–alumina porous capillary supports for filtration application. J. Membr. Sci..

[2] Shkrabina R.A., Boneckamp B., Pex P., Veringa H., Ismagilov Z.R. (1995). Porous structure of alumina ceramic support for gas separation membranes, II. Study of porous structure of ceramic composition. React. Kinet. Catal. Lett..

[3] Dong Y., Chen S., Zhang X., Yang J., Liu X., Meng G. (2006). Fabrication and characterization of low cost tubular mineral-based ceramic membranes for micro-filtration from natural zeolite. J. Membr. Sci..

[4] Bao Q., Dong W., Zhou J.E., Wang Y., Liu Y. (2015). Effects of pore former on properties of alumina porous ceramic for application in micro-filtration membrane supports. Key Eng. Mater..

[5] Zhou J.E., Yang Y., Chang Q., Wang Y., Ke Y., Bao Q. (2014). Effect of particle size gradients of alumina powders on the pore size distribution and bending strength of ceramic membrane supports. J. Synth. Cryst..

[6] Chang Q., Yang Y., Zhang X., Wang Y., Zhou J., Wang X., Cerneaux S., Dong Y. (2014). Effect of particle size distribution of raw powders on pore size distribution and bending strength of Al_2_O_3_ microfiltration membrane supports. J. Eur. Ceram. Soc..

[7] Goei R., Lim T.T. (2014). Asymmetric TiO_2_, hybrid photocatalytic ceramic membrane with porosity gradient: Effect of structure directing agent on the resulting membranes architecture and performances. Ceram. Int..

[8] Wei L., Huang Y. (2015). Preparation of porous TiO_2_/stainless-steel membranes by carbon assisted solid-state particle sintering. J. Inorg. Mater..

[9] Baig M., Patel F., Alhooshani K., Muraza O., Wang E., Laoui T. (2015). In-situ aging microwave heating synthesis of LTA zeolite layer on mesoporous TiO_2_ coated porous alumina support. J. Cryst. Growth.

[10] Kruft J., Bruck H., Shabana Y. (2008). Effect of TiO_2_ nanopowder on the sintering behavior of nickel–alumina composites for functionally graded materials. J. Am. Ceram. Soc..

[11] Liu X., Zheng J., Li C., Wu M., Jia D., Li Y. (2014). Optimization of alumina powder preparation conditions for ceramic membrane support sintering. J. Aust. Ceram. Soc..

[12] Wu Z., Zang L., Chen Y., Xie Y. (2001). Gelcasting of Al_2_O_3_ with TiO_2_ added: the effects of sintering aid and dispersant. Chin. J. Process. Eng..

[13] Yang Y., Zhou J.E., Wang Y., Chang Q., Yang K. (2015). Effect of nano-TiO_2_ on sintering process of alumina porous ceramic membrane support. J. Synth. Cryst..

[14] Chang Q., Wang Y., Cerneaux S., Zhou J.E., Zhang X., Wang X. (2014). Preparation of microfiltration membrane supports using coarse alumina grains coated by nano TiO_2_, as raw materials. J. Eur. Ceram. Soc..

[15] Qi H., Fan Y., Xing W., Winnubst L. (2010). Effect of TiO_2_ doping on the characteristics of macroporous Al_2_O_3_/TiO_2_ membrane support. J. Eur. Ceram. Soc..

[16] Li D., Zhu Q., Cui S. (2012). Preparation and characterization of circular plate-shaped porous alumina ceramic membrane support. J. Environ. Eng..

[17] Wang Y., Chen G., Wang Z., Liu J., Luo P. (2018). Improvement of microcracks resistance of porous aluminium titanate ceramic membrane support using attapulgite clay as additive clay as additive. Ceram. Int..

[18] Oun A., Tahri N., Mahouche-Chergui S., Carbonnier B., Majumdar S., Sarkar S. (2017). Tubular ultrafiltration ceramic membrane based on titania nanoparticles immobilized on macroporous clay-alumina support: Elaboration, characterization and application to dye removal. Sep. Purif. Technol..

[19] Monash P., Pugazhenthi G. (2011). Effect of TiO_2_ addition on the fabrication of ceramic membrane supports: A study on the separation of oil droplets and bovine serum albumin (BSA) from its solution. Desalination.

[20] Bissett H., Zah J., Krieg H.M. (2008). Manufacture and optimization of tubular ceramic membrane supports. Powder Technol..

